# Infection of Xenotransplanted Human Cell Lines by Murine Retroviruses: A Lesson Brought Back to Light by XMRV

**DOI:** 10.3389/fonc.2013.00156

**Published:** 2013-06-17

**Authors:** Heidi A. Hempel, Kathleen H. Burns, Angelo M. De Marzo, Karen S. Sfanos

**Affiliations:** ^1^Department of Pathology, Johns Hopkins University School of Medicine, Baltimore, MD, USA; ^2^Institute of Genetic Medicine, Johns Hopkins University School of Medicine, Baltimore, MD, USA; ^3^The Sidney Kimmel Comprehensive Cancer Center, Johns Hopkins University School of Medicine, Baltimore, MD, USA

**Keywords:** gammaretrovirus, cell line, xenotransplantation, XMRV, cancer

## Abstract

Infection of xenotransplanted human cells by xenotropic retroviruses is a known phenomenon in the scientific literature, with examples cited since the early 1970s. However, arguably, until recently, the importance of this phenomenon had not been largely recognized. The emergence and subsequent debunking of Xenotropic Murine leukemia virus-Related Virus (XMRV) as a cell culture contaminant as opposed to a potential pathogen in several human diseases, notably prostate cancer and Chronic Fatigue Syndrome, highlighted a potential problem of murine endogenous gammaretroviruses infecting commonly used human cell lines. Subsequent to the discovery of XMRV, many additional cell lines that underwent xenotransplantation in mice have been shown to harbor murine gammaretroviruses. Such retroviral infection poses the threat of not only confounding experiments performed in these cell lines via virus-induced changes in cellular behavior but also the potential infection of other cell lines cultured in the same laboratory. Thus, the possibility of xenotropic retroviral infection of cell lines may warrant additional precautions, such as periodic testing for retroviral sequences in cell lines cultured in the laboratory.

## The Search for Retroviruses Associated with Human Disease

The considerable role of retroviruses in animal disease is well-established. Knowing this, retroviral causes for many human diseases have been earnestly sought. Four notable retroviral pathogens in this respect are human T-lymphotropic virus type 1 and type 2 (HTLV-1and HTLV-2), and human immunodeficiency virus type 1 and type 2 (HIV-1 and HIV-2). HTLV-1 has been shown to be causal in both adult T-cell leukemia and neurological disease (Voisset et al., 2008; Mauclère et al., 2011). HTLV-2 has been associated with TSP/Ham-like diseases, as well as lymphocytosis (Voisset et al., 2008; Mauclère et al., 2011). HIV-1 and 2 are both associated with acquired immunodeficiency syndrome (AIDS). Additional retroviruses have been implicated in a variety of human diseases, including many cancers, neurological disorders, and inflammatory diseases. However, establishing causation has proved exceedingly difficult for a number of reasons, including the inability to replicate results (Voisset et al., 2008). Despite these difficulties, the search for human disease-causing retroviruses has remained a hot field, and much excitement can be generated over new discoveries. Such was the case in the discovery of Xenotropic Murine leukemia virus-Related Virus (XMRV), in which a study describing a possible connection between this retrovirus and prostate cancer (PCa) resulted in a hail-storm of studies looking to connect XMRV to a number of additional human diseases, most notably to chronic fatigue syndrome (CFS).

## The Story of XMRV

Xenotropic Murine leukemia virus-Related Virus was first described in 2006, when viral sequences were detected in RNA samples isolated from PCa tissues (Urisman et al., 2006) (the Urisman et al., reference appeared in a March 2006 issue of PLoS Pathogens, and was fully retracted by the editors in September 2012). Several follow-up studies, however, did not detect XMRV in PCa patient samples (Fischer et al., 2008; Sfanos et al., 2008; Hohn et al., 2009; Aloia et al., 2010; Sakuma et al., 2011; Switzer et al., 2011). In addition, studies conducted in 2009 (Schlaberg et al., 2009) and 2010 (Arnold et al., 2010; Danielson et al., 2010) supporting the presence of XMRV in PCa had apparent contradictions to the original report as well as to each other, including discrepancies in the presence of virus in tumor versus benign tissues and discordance in cellular localization (reviewed in Sfanos et al., 2012). Despite this, the possibility of a viral cause to PCa proved to be an exciting prospect, prompting follow-up studies in PCa as well as other diseases. Studies exploring the possibility of XMRV involvement in different cancers, autoimmune diseases, HIV, and even autism were reported, with differing results (McCormick et al., 2008; Barnes et al., 2010; Cornelissen et al., 2010; Jeziorski et al., 2010; Satterfield et al., 2010; Balada et al., 2011; Gray et al., 2011; Lintas et al., 2011; Schmitt et al., 2011; Tang et al., 2011; Waugh et al., 2011; Maggi et al., 2012). Of particular concern was the detection of XMRV in sera collected from patients with CFS (a disorder of unknown etiology characterized by persistent fatigue) as well as several healthy controls (Lombardi et al., 2009) (the Lombardi et al. reference appeared in an October 2009 issue of *Science*, and was fully retracted by the editors in December 2011). With this study came serious implications for the safety of the nation’s blood supply, prompting immediate follow-up studies that, again, contradicted the original report (Erlwein et al., 2010; Groom et al., 2010). An additional study, initially touted as the CFS “confirmatory” study, reported the detection of murine leukemia virus (MLV) sequences in CFS patient samples. These sequences were not XMRV however, but polytropic endogenous MLV sequences that are present in the mouse genome – a finding that largely implicated murine DNA contamination of the samples (Lo et al., 2010) (the Lo et al. reference appeared in a September 2010 issue of *The Proceedings of the National Academy of Sciences*, and was retracted by the authors in January 2012). With so many contradictions building in the literature, the role of XMRV in human disease was brought into serious doubt.

The debunking of XMRV as a cause of human disease was largely threefold. Firstly, mouse genomic DNA was found to contain endogenous retrovirus sequences that amplified with “XMRV-specific” primers in PCR assays (Hue et al., 2010). In 2010, a highly sensitive PCR technique was developed to detect contaminating murine mitochondrial cytochrome oxidase and intracisternal A particle (IAP) sequences in DNA samples (Oakes et al., 2010). Looking at a series of clinical samples, every sample that had been identified as XMRV positive was also positive for murine DNA contamination (Oakes et al., 2010; Robinson et al., 2010). In addition, many commercial reagents used to study XMRV were found to be similarly contaminated with murine DNA, accounting for other false-positive results in XMRV studies (Sato et al., 2010; Erlwein et al., 2011; Tuke et al., 2011).

Next, the results of a joint study termed the Blood XMRV Scientific Research Working Group (SRWG) were published (Simmons et al., 2011). In this study, blinded samples of XMRV-spiked positive controls, clinical samples previously reported to be infected, and negative controls were sent to nine different labs for XMRV detection by various methods. Two of these labs, which had also been involved in the original XMRV-CFS study, detected XRMV in both the clinical samples and the negative controls. Another lab that had previously reported XMRV in clinical samples, only reported presence of the virus in the positive control samples. These results strongly suggested issues with assay reproducibility and false positives due to contamination (Simmons et al., 2011).

The final major blow to XMRV as a root of human disease lies in a study in which the recombinant origin of XMRV was described (Paprotka et al., 2011). In this study, Paprotka et al. demonstrated that XMRV originated by recombination between two distinct endogenous MLVs found in nude mouse strains, PreXMRV-1 and PreXMRV-2, that were likely recombined during the xenotransplantation of the PCa cell line CWR22Rv1 through nude mice. CWR22Rv1 subsequently produced XMRV and is thought to be the singular source of the laboratory contaminant. Supporting this conclusion, essentially all XMRV sequences isolated from clinical samples are the same composite sequence, with its unique signature of switch positions between PreXMRV-1 and PreXMRV-2 (Paprotka et al., 2011). Lastly, in 2012, the results of a National Institute of Allergy and Infectious Diseases (NIAID)-sponsored multicenter, blinded study were published, affirming that XMRV is not present in CFS patient samples (Alter et al., 2012).

## Murine Endogenous Retroviruses in Human Cancer Cell Lines

The lesson of XMRV is a potentially important one. The fact that XMRV arose from xenotransplantation (i.e., xenografting) of CWR22Rv1 through mice – a very common practice when developing cancer cell lines – is particularly concerning. Although most researchers appear to be unaware of the potential contamination threat (Zhang et al., 2011), the infection of xenografted human cell lines with xenotropic retroviruses is well-established in the literature, dating back to the early 1970s (Takeuchi et al., 2008; Sfanos et al., 2011). In 1972, an endogenous feline retrovirus, RD114, was found to have infected human rhabdomyosarcoma cells that had been xenotransplanted through a fetal kitten brain (McAllister et al., 1972). In 1973, there was a similar finding in that a murine type-C retrovirus was isolated from rhabdomyosarcoma cells that had been xenotransplanted through NIH Swiss mice (Todaro et al., 1973). However, the implications of these and other findings have arguably not been fully realized. To date, and particularly after the XMRV controversy, several additional reports highlight the widespread issue of xenotropic MLV (XMLV) contamination of xenotransplanted cell lines (Table 1).

**Table 1:** **Reports of XMLV infection of cancer cell lines**.

Cell line	Origin	Xenografted?^±^	Virus (Ref.)*
CWR22Rv1	Prostate cancer	Yes	XMRV (Knouf et al., 2009)
LAPC4	Prostate cancer	Yes	*Bxv*-1/N417 MLV, MLV SP(B) 1-2 (Sfanos et al., 2011;Zhang et al., 2011)
VCaP	Prostate cancer	Yes	*Bxv*-1 MLV (Sfanos et al., 2011)
EKVX	NSCLC	Yes	DG-75 MLV (Hue et al., 2010;Sfanos et al., 2011)
DG-75	Burkitt’s lymphoma	No	DG-75 MLV (Raisch et al., 2003)
CAK1	Pancreas cancer	Yes	Unidentified XMLV (Zhang et al., 2011)
LX48	SCLC	Yes	Unidentified XMLV (Zhang et al., 2011)
LX47	SCLC	Yes	Unidentified XMLV (Zhang et al., 2011)
NCI-N417	SCLC	Yes	N417 MLV (Zhang et al., 2011)
1065met	SCLC	Yes	Unidentified XMLV (Zhang et al., 2011)
Jurkat J6	T cell leukemia	Unknown	Unidentified XMLV (Takeuchi et al., 2008)
SK-MEL-25	Melanoma	No	Unidentified XMLV (Deichmann et al., 2005)
SK-MEL-28**	Melanoma	No	Unidentified XMLV (Deichmann et al., 2005)
MEL-JUSO	Melanoma	No	Unidentified XMLV (Deichmann et al., 2005)
MML-1	Melanoma	No	Unidentified XMLV (Deichmann et al., 2005)
A2780	Ovarian cancer	No	Unidentified XMLV (Hue et al., 2010)
BHY	Squamous cell carcinoma	No	Unidentified XMLV (Hue et al., 2010)
CoCM-1	Colon cancer	No	Unidentified XMLV (Hue et al., 2010)
Daudi	Burkitt’s lymphoma	No	Unidentified XMLV (Hue et al., 2010)
IMR-5	Neuroblastoma	No	Unidentified XMLV (Hue et al., 2010)
MUTZ-1	Myeloid leukemia	No	Unidentified XMLV (Hue et al., 2010)
S-117	Thyroid sarcoma	Unknown	Unidentified XMLV (Hue et al., 2010)
TYK-nu	Ovarian cancer	Yes	Unidentified XMLV (Hue et al., 2010)

*±For cell lines that were not xenografted in mice, it is possible that cross-contamination from infected xenografted lines occurred, or that studies involving xenotransplantation were performed with these lines, resulting in XMLV infection*.

*Reference given is for viral genome sequence, if available.

**The SK-MEL-28 cell line was found to be negative for XMLVs in Hue et al. (2010) and Sfanos et al. (2011).

Hue et al. (2010) screened 411 cell lines from the Catalog of Somatic Mutations in Cancer (COSMIC) collection by XMRV- and XMLV-specific PCR. Nine of these lines were found to contain contaminating XMLVs (see Table 1) (Hue et al., 2010). Likewise, in 2011, Sfanos et al. (2011) published a study whereby using immunohistochemistry (IHC) and PCR techniques, they tested 72 cell lines, including 58 of the 60 human cancer cell lines commonly used in anti-cancer drug screens and maintained at the NCI-Frederick (NCI-60) for the presence of XMRV or other XMLVs. Three cell lines, in addition to CWR22Rv1, were identified as being infected with XMLV species. Two of these were other PCa cell lines, namely LAPC4 and VCaP, while the third was EKVX, a non-small-cell lung carcinoma (NSCLC) cell line. Out of the four infected lines, only CWR22Rv1 was infected with XMRV. LAPC4 and VCAP were found to harbor a virus 99% similar to a previously described XMLV, *Bxv*-1 (also known as XMV43). EKVX was found to be infected with an XMLV that is 98% similar to a retrovirus previously isolated from the B lymphoblastic cell line, DG-75. Finally, Sfanos et al. (2011) showed, using a novel viral infectivity assay (Aloia et al., 2013), that the XMLVs infecting LAPC4 and VCaP were replication competent, while the virus infecting EKVX had very low infectivity.

Concurrent with the Sfanos et al. study was a report by Zhang et al. (2011) that sought to determine the frequency of XMLV infection of xenografted cancer cell lines and the frequency of XMLV spread to non-xenograft-derived lines maintained in facilities that also harbor xenografted lines. The presence of MLV sequences in 26 xenografted cell lines gathered from seven independent laboratories was assayed by validated Taqman PCR assays. Three cell lines were omitted due to the detection of murine DNA contamination. Of the remaining 23 cell lines, 6 lines (1065met, NCI-N417, LX47, LX48, CAK1, LAPC4, see Table 1) were found to contain MLV sequences. The viral genomes found in two xenografted cell lines (NCI-N417 and LAPC4) were then characterized. Interestingly, there were previous hints that the NCI-N417 small cell lung cancer (SCLC) cell line harbored an XMLV. This line was previously reported to express a homologous transcript to the v-*fms* protooncogene via Northern blot analysis (Kiefer et al., 1987). However, the vector used as a probe to detect v-*fms* by Northern blot contained a significant amount of the *pol* gene of the Susan McDonough strain of feline sarcoma virus. It was subsequently discovered that the viral portion of the probe contained significant sequence homology to AKV MLV and that the positive signal by Northern blot analysis was not due to the presence of v-*fms*, but due to the presence of MLV transcripts in the NCI-N417 cell line (Walker et al., 1989). The viral genome isolated from NCI-N417 has now been fully sequenced and deposited in the GenBank sequence database as MLV strain N417 (GenBank Accession number HQ246218) (Zhang et al., 2011).

Zhang et al. also sequenced two PCR products covering the MLV *gag* and *env* gene regions from the LAPC4 cell line. These sequences were found to be 100% identical to the N417 MLV genome sequenced from the NCI-N417 cell line (Zhang et al., 2011). These sequences are also 100% identical to the full viral genome sequenced from the LAPC4 cell line by Sfanos et al. (2011) (GenBank Accession number JF908816). As previously mentioned, Sfanos et al. reported that the virus in LAPC4 was 99% similar to the known XMLV *Bxv*-1. The N417 virus is also 99% similar to *Bxv*-1, and the full-length virus sequenced by Sfanos et al. from LAPC4 only differs from the N417 virus genome by three bases. Zhang et al. (2011) also detected the presence of an additional virus with sequence similarity to MLV SP(B) 1–2 (GenBank Accession number AY349140) in the LAPC4 line.

Finally, the cell culture supernatant of NCI-417, LAPC4, and 1065met were evaluated for viral infectivity, with the results showing infective capabilities in all three cell lines (Zhang et al., 2011).

These studies bring to light a potentially serious problem lurking within *in vitro* research. The danger is not isolated to the wasted resources in researching potential disease etiology of contaminating retroviruses. There is also significant evidence to suggest that the unknown presence of XMLVs in commonly used cancer cell lines can have a confounding role in scientific research studies using these contaminated lines.

## Infection by XMLVs Leading to Changes in Cellular Behavior

There are several potential mechanisms by which XMLVs may modify the biological properties of a cell line, including viral integration/insertional mutagenesis and alterations to *in vivo* immunogenicity. Several recent studies on XMRV have highlighted the potential for XMLVs to induce changes in cellular behavior, thus posing a potentially serious problem in the form of confounding results of studies using infected cell lines.

In their study exploring the possible pathogenic role of XMRV in PCa, Pandhare-Dash et al. (2012) clearly demonstrated that infection of PCa cell lines by XMRV can induce significant changes in cellular behavior. Two human PCa cell lines (LNCaP and PC3) were infected with an XMRV strain isolated from a chronically infected LNCaP line, and tested for changes in proliferation, anchorage-independent growth, invasiveness, and matrix metalloproteinase (MMP) production (Pandhare-Dash et al., 2012). Both proliferation and anchorage-independent growth increased in LNCaP cells, but not in PC3 cells. However, invasiveness and production of MMP-2 and MMP-9 were increased in both cell lines. Furthermore, p27^*Kipl*^ protein levels were analyzed, and infected LNCaP cells were found to have lower protein expression than uninfected cells. Infected LNCaP cells were also found to have a higher percentage of cells in S phase of the cell cycle than uninfected cells. Upon further analysis, Pandhare-Dash et al. found that LNCaP cells infected with XMRV upregulated miR-221 and miR-222 (which inhibit p27^*Kipl*^ expression) compared to uninfected cells, presenting a mechanism by which infected cells could be downregulating p27^*Kipl*^ and thus promoting progression through the G_1_-S transition of the cell cycle. XMRV infection of the androgen receptor (AR) negative DU145 cell line did not induce changes in cellular proliferation or p27^*Kipl*^ levels, but did induce slightly higher levels of MMP activity versus uninfected DU145 cells. Thus, Pandhare-Dash et al. demonstrated behavioral changes in three different PCa cell lines due to infection with XMRV.

Stieler et al. (2012) published a report aimed at determining whether the XMRV-infected CWR22Rv1 cell line is a suitable PCa model. CWR22Rv1 lines that expressed significantly reduced numbers of viral transcripts were developed using anti-viral long terminal repeat (LTR) shRNA. Xenografted tumors established with these lines weighed less, were significantly more necrotic, and showed a significant reduction in blood vessel formation compared to unaltered CWR22Rv1 cells (Stieler et al., 2012). *In vitro*, the virus-reduced cells had significantly different cytokine expression patterns than the unaltered CWR22Rv1 cells, with decreased expression of OPN, TIMP2, IL-13, and CXCL14, and increased expression of HGF. Moreover, *de novo* infection of primary prostate stromal fibroblasts (PrSc cells) with replication-competent XMRV also resulted in cytokine expression changes, with increased expression of GROa and decreased expression of IL-13 and TIMP1. The group also tested whether XMRV-infected cells could affect the migration of nearby cells in a paracrine manner. Interestingly, uninfected LNCaP cells separated from infected PrSc cells by a permissive membrane showed a statistically significant increase in migration. In addition, stimulation of human mammary epithelial (HMEC) cells cultured in Matrigel with supernatant from XMRV-infected PrSc cells showed a significant increase in tube formation, suggesting that infection with XMRV also has a direct effect on angiogenesis. In all, Stieler et al. (2012) showed that infection with XMRV not only affected cellular behavior *in vivo* and *in vitro* and in two cellular systems (CWR22Rv1 and PrSc cells), but that those changes in cellular behavior can also have paracrine effects on non-infected cells.

Finally, Mohan et al. (2012) recently reported global changes in microRNA (miRNA) expression upon XMRV infection of LNCaP and DU145 cells. Furthermore, this group reported consistent downregulation of miR-193a-3p and changes in miRPlus-E1245 levels that were specific to XMRV infection across four different cell types (DU145, LNCaP, peripheral blood lymphocytes, and monocyte-derived macrophages).

## XMLV Contamination of Non-Xenotransplanted Cell Lines

Since many of the XMLV-infected cell lines in both the Sfanos et al. (2011) and Zhang et al. (2011) studies were found to be replication competent, the potential for cross-contamination of other cell lines grown in the same facilities is considerable. Indeed, Sfanos et al. (2011) reported that XMRV-negative cell lines that were being cultured in the laboratory at the same time as CWR22Rv1 had become contaminated. Similarly, Zhang et al. (2011) detected XMLV infection of 13 out of 78 non-xenografted cell lines from five different laboratories that were also culturing XMLV-infected xenografted cell lines. In contrast, all 50 cell lines gathered from non-xenograft culturing labs were XMLV-free. The laboratories involved in both studies were using standard BSL2 aseptic technique, highlighting the infective potential of these XMLVs. Therefore, the dangers of XMLV contamination of human cell lines is not limited to xenografted lines, but potentially extends to all additional cell lines being cultured alongside infected lines. If contamination occurs and is not detected, there is significant potential for spreading of the retrovirus, even across laboratories sharing samples.

## Concluding Remarks

Excitement quickly mounted when the first paper linking XMRV to PCa was published, gaining sufficient momentum to fuel many studies looking to link XMRV to many different human diseases. The implications of XMRV having since been recognized as a cell culture contaminant go beyond elimination of its potential role in human disease. The discovery and subsequent debunking of XMRV as a human pathogen brought back to light the issue of XMLV infection of xenotransplanted cell lines. This includes potentially artifactual experimental results due to virus-induced changes in cellular behavior, as well as the cross-contamination of uninfected cell lines grown in the same laboratory. With these dangers in mind, precautions, such as routine testing for XMLVs in human cell lines developed by xenotransplantation, or any cell lines cultured in laboratories concurrently growing xenotransplanted or known XMLV-infected cell lines, may warrant serious consideration.

## Conflict of Interest Statement

Angelo M. De Marzo is currently an employee of Predictive Biosciences Inc., who also holds a part-time adjunct appointment at the Johns Hopkins University School of Medicine. However, no funding or other support was provided by the company for any of the work in this manuscript. The terms of the relationship between Angelo M. De Marzo and Predictive Biosciences are managed by the Johns Hopkins University in accordance with its conflict of interest policies.
